# EXPLORING THE EFFICACY OF ARTIFICIAL INTELLIGENCE IN DELIVERING HOSPICE INFORMATION

**DOI:** 10.1093/geroni/igad104.3334

**Published:** 2023-12-21

**Authors:** Jeanette Ross, Alexander Dentchev, Elizabeth Reilly, Sandra Sanchez-Reilly

**Affiliations:** South Texas Veterans Health Care System, San Antonio, Texas, United States; Saint Mary’s Hall, San Antonio, Texas, United States; Health Careers High School, San Antonio, Texas, United States; South Texas Veterans Health Care System, San Antonio, Texas, United States

## Abstract

**Background:**

Artificial Intelligence Chatbots (AIC) are swiftly gaining popularity due to their language model capability to address user queries by analyzing extensive amounts of available data and generating human-like responses. Objective: To analyze the accuracy of AIC in responding to inquiries related to hospice services and benefits. Additionally, the goal is to assess the user-friendliness of AIC in comparison to traditional web search engines.

**Methods:**

AICs, namely ChatGPT and Bard, were subjected to testing through a series of questions and scenarios pertinent to hospice services (such as its definition, eligibility criteria, types of provided services, and specific care scenarios). Recorded responses from the AICs were subsequently evaluated for accuracy and quality by experts in geriatric palliative care.

**Results:**

Both ChatGPT and Bard provided mostly accurate responses to fundamental questions and uncomplicated scenarios regarding hospice. These encompassed explanations about prognosis, interdisciplinary teams, and basic services offered. While ChatGPT did not furnish articles or source references, it did offer occasional medical advice. On the other hand, Bard issued medical information warnings and occasionally included visual aids like pictures, diagrams, and website links. When faced with the same set of questions, conventional web search engines often supplied links to webpages that did not invariably contain specific answers to the queries.

**Conclusions:**

The AICs can deliver predominantly accurate, generic information about hospice in a conversational and comprehensible manner. Future research could delve into how AICs would respond to more personalized and specific inquiries that could be posed by patients or caregivers.

